# Preparation of Excipient-Free Molnupiravir Microparticles for Pulmonary Drug Delivery and Process Evaluation via Machine Learning

**DOI:** 10.3390/pharmaceutics18101290

**Published:** 2026-10-10

**Authors:** Juncheng Hu, Yucheng Huang, Jiahao Luo, Jiayi Wang, Haoyang Chen, Minghao Lian, Ning Xue, Xiang Luo, Kaiqi Shi, Bin Dong, Hanhan Li

**Affiliations:** 1 School of Artificial Intelligent Medicine, Guilin Medical University, Guilin 541199, China; jc.hu@glmc.edu.cn; 2 Department of Pharmaceutical and Environmental Engineering, China Pharmaceutical University, Nanjing 211198, China; 3 Department of Computer Science, University of Nottingham Ningbo China, Ningbo 315100, China; ning-xue@nottingham.edu.cn; 4 Department of Chemical and Environmental Engineering, the University of Nottingham Ningbo China, Ningbo 315100, China; xiang-luo@nottingham.edu.cn; 5 Suzhou Inhal Pharma Co., Ltd., Suzhou 215000, China; skq@inhalpharma.com; 6 Engineering Research Center for Smart Pharmaceutical Manufacturing Technologies, Ministry of Education, Nanjing 210009, China; 7 School of Basic Medicine and Clinical Pharmacy, China Pharmaceutical University, Nanjing 211198, China

**Keywords:** molnupiravir, micropowder, machine learning, pulmonary drug delivery

## Abstract

**Background/Objective:** Molnupiravir (EIDD-2801) was originally designed to treat influenza A virus and showed broad-spectrum antiviral activity, including against influenza virus, Ebola virus, coronavirus and other viruses. **Methods:** In this study, molnupiravir microparticles were firstly prepared by spray freeze-drying (SFD) and jet milling (JM) methods. Meanwhile, machine learning methodology was applied to evaluate the influence of process parameters such as feeding rate, concentration, and nebulization pressure on powder properties. **Results:** According to SEM images, the microparticles prepared by SFD presented a porous and near-spherical structure. Moreover, the fine particle fraction (FPF) and mass median aerodynamic diameter (MMAD) of the optimal inhalable powder reached up to 70.19 ± 2.06% and 2.80 ± 0.16 μm, proving the excellent aerodynamic performance. **Conclusions:** The molnupiravir micropowder showed preliminary short-term storage behavior after storage for 28 days at 25 °C and 60% RH.

## 1. Introduction

As a prodrug and nucleotide analog, molnupiravir possesses broad-spectrum antiviral activity against multiple viruses, including influenza virus, Ebola virus, coronavirus and other viruses [1,2]. Molnupiravir was originally designed for the treatment of influenza A virus infection; it can be hydrolyzed and converted to the active metabolite N^4^-hydroxycytidine (EIDD-1931), which is further phosphorylated into 5′-triphosphate. As a substrate of viral RNA-dependent RNA polymerases, EIDD-1931-5′-triphosphate can participate in the replication of viral RNA, leading to catastrophic errors in replication [3,4]. Recently, numerous studies have verified the excellent viral inhibition of molnupiravir in animal models with SARS-CoV-2 infection in multiple animal models, including rhesus macaques, ferrets, hamsters, and mice [5,6,7]. Furthermore, benefiting from its high oral bioavailability, metabolic stability and low toxicity, molnupiravir has been approved for clinical use in China, the United Kingdom, and the United States [8,9,10,11].

Pulmonary drug delivery (PDD) is a targeted drug delivery strategy that enables direct drug administration to the lungs via specialized inhalation devices [12,13,14]. Compared with conventional administration routes, PDD effectively circumvents hepatic first-pass metabolism and improves patient adherence to treatment [15,16]. Due to the unique structure of the lung, PDD has exhibited distinct therapeutic advantages. Firstly, the drug absorption efficiency can be increased by the large surface area of the human lung (60–130 m^2^) and the higher blood flow and total blood volume in the lung (reaching up to 5–6 L/min and 0.5 L) [17]. Therefore, the blood in the lungs will be completely exchanged every 5 s, and drugs can be transported directly to the lesion efficiently and rapidly. Moreover, the total number of human alveoli is up to 5.6 × 10^8^, and the distance between alveolar epithelial cells and the capillary wall is about 1 μm [18]. Undoubtedly, the good permeability of alveolar epithelial cells and the huge number of alveoli promote the absorption of drugs, further enhancing the efficacy and bioavailability.

The physicochemical properties of inhalable powders play a vital role in determining the deposition effect of particles in the lung, which further affects the clinical therapeutic outcome [19,20]. Specifically, particles with an aerodynamic diameter range of 1–5 μm can achieve optimal deep lung deposition. In contrast, particles larger than 5 μm are predominantly deposited in the oropharynx region and subsequently swallowed into the gastrointestinal tract, while particles smaller than 1 μm will not be able to settle in the lung periphery and are rapidly exhaled with breathing [21]. In addition to appropriate particle diameters, the physico-chemical stability of inhalable powders is essential for maintaining sustained drug efficacy under the high-temperature and high-humidity microenvironment of the lungs [22]. Moreover, inhalable powders with superior dispersibility and flowability can minimize particle retention in inhaler devices, thereby improving the emitted dose fraction and delivery efficiency [23].

Currently, artificial intelligence (AI) has emerged as a powerful technology in pharmaceutical manufacturing and drug delivery research [24,25]. In 2023, Murray et al. highlighted that data-driven modeling holds transformative potential for pharmaceutical formulation development [26]. In 2026, Ros et al. illustrated that surrogate modeling, Bayesian optimization, active learning and multi-objective optimization were key developmental frontiers for formulation manufacturing [27]. Moreover, Vora et al. have collectively demonstrated that machine learning (ML) has been extensively implemented for formulation screening, process optimization, quality attribute prediction, and intelligent pharmaceutical manufacturing [28].

To the best of our knowledge, there is no research on preparing excipient-free molnupiravir microparticles for pulmonary drug delivery. Therefore, molnupiravir powders were prepared in this work in a controlled manner using spray freeze-drying (SFD) and jet milling (JM) methods. The physicochemical properties of as-prepared particles were systematically investigated, while the relevant process parameters were optimized with machine learning. The results indicated the great potential of SFD in the preparation of inhalable molnupiravir microparticles with a porous and near-spherical structure, which exhibited good aerosol performance.

## 2. Materials and Methods

### 2.1. Materials

The materials used in this study are as follows: molnupiravir (99%, Shandong Sihuan Pharmaceutical, Dezhou, China), chromatographic acetonitrile (Honeywell, Charlotte, NC, USA), glycerol (Shanghai Macklin Biochemical Co., Ltd., Pudong, China), methanol (Shanghai Macklin Biochemical Co., Ltd., Pudong, China), dimethyl sulfoxide (Energy Chemical, Zhangjiang, China), chloral hydrate (Shanghai Macklin Biochemical Co., Ltd., Pudong, China), size 3 gelatine capsules (Jiangxi Fumeitai, Yichun, China), MTT (Beijing InnoChem Science & Technology Co., Ltd., Beijing, China), TNF-α Elisa kit (Multi Sciences, Hangzhou, China), and IL-6 Elisa kit (Multi Sciences, Hangzhou, China).

### 2.2. Preparation of Molnupiravir Powder

In this study, molnupiravir powder was prepared using spray freeze-drying and jet milling methods. Firstly, samples containing molnupiravir were nebulized and frozen by a spray freezer (YC-01, Pilotech, Shanghai, China), and then freeze-dried using a freeze dryer (LGJ-10D, Beijing Sihuan Qihuang Technology Co., Ltd., Beijing, China) for 48 h under pressurized conditions below 20 Pa. A set of single-factor experiments with nebulization pressure, feeding rate and concentration as variables were designed, and the experimental preparation conditions are shown in Table S1.

Secondly, molnupiravir API was also micronized by a jet mill (MC Decjet 30, Dec Group, Ecublens, Switzerland) with feeding pressure of 8 bar and crushing pressure of 8 bar.

### 2.3. Size Distribution

The particle size distribution (PSD) of samples was measured by a laser diffraction particle sizer (HELOS/RODOS, SYMPATEC GmbH Co. Ltd., Clausthal-Zellerfeld, Germany). Approximately 10 mg of sample was loaded into a test tube and tested with an R5 (4.5–875 μm) lens. Each sample was measured in triplicate, and its volume particle size was recorded three times in parallel to record its tenth percentile (*D*_10_), median (*D*_50_), ninetieth percentile (*D*_90_) and volume median diameter (*D*_V_). The value of span can be calculated using Equation (1) [29].

(1) $$\mathrm{Span}=\frac{D_{90}-D_{10}}{D_{50}}$$

### 2.4. Measurement of Bulk Density (Ρ_a_), Tap Density (Ρ_b_), Carr Index (Ci) and Hausner Ratio (Hr)

The density of samples was determined using a graduated cylinder. Powder was accurately weighed in a 5 mL graduated cylinder; its mass was recorded as *m*, and the volume was recorded as *V*_1_ without applying any external force. Then the powder was continuously tapped until its volume remained constant. The volume was recorded as *V*_2_. The values of bulk density (*ρ*_a_), tap density (*ρ*_b_), Carr index (CI) and Hausner ratio (HR) were calculated according to the following Equations (2) to (5).

(2) $$\rho_{a}=\frac{m}{V_{1}}$$

(3) $$\rho_{b}=\frac{m}{V_{2}}$$

(4) $$\mathrm{CI}\%=(\frac{\rho_{b}-\rho_{a}}{\rho_{b}})\times 100\%$$

(5) $$\mathrm{HR}=\frac{\rho_{b}}{\rho_{a}}$$

### 2.5. Measurement of Aerodynamic Equivalent Diameter

Based on the value of tap density and particle size, the approximate theoretical value of the aerodynamic equivalent diameter (*D*_ae_) of the powder was calculated according to the following Equation (6). *D*_ae_ refers to the diameter of a unit-density spherical particle that has the same sedimentation rate [30,31].

(6) $$D_{\mathrm{ae}}=D_{V}\sqrt{\frac{\rho_{b}}{\rho_{0}x}}$$

*x* represents the morphological coefficient of the powder; for approximately spherical particles, *x* = 1. *ρ*_0_ represents unit density (1 g/cm^3^).

### 2.6. X-Ray Diffraction (Xrd)

The crystallinity of samples was measured by the XRD diffractometer (D8 Discover, Bruker; Billerica, MA, USA). Samples were scanned at a voltage of 40 kV and subjected to Cu radiation in the range of 50 to 90° (2θ). The step size and scanning speed were 0.02 and 10°/min.

### 2.7. Morphology

The morphology of the samples was measured using a scanning electron microscope (SEM) (S-4800, Hitachi; Tokyo, Japan) at an accelerating voltage of 5.0 kV. The samples were dispersed onto a conductive tape before the analysis.

### 2.8. Fourier-Transform Infrared Spectroscopy (Ftir)

The infrared spectra of samples were measured by an FTIR spectrometer (Nicolet iS5, Thermo Scientific, Waltham, MA, USA) with an ATR accessory. About 10 mg of the sample was weighed and analyzed; the transmission was recorded from an average of 32 scans over 400–4000 cm^−1^.

### 2.9. Thermogravimetric Analysis (Tg)

In order to test the thermal stability, about 10 mg of the sample was accurately weighed in a ceramic crucible and placed in the TG thermogravimetric analyzer (TG 209 F3 Tarsus, NETZSCH, Selb, Germany). The samples were heated from room temperature to 500 °C at a heating rate of 40 k/min.

### 2.10. Differential Scanning Calorimetry (DSC)

A differential scanning calorimeter (DSC 214 Polyma, NETZSCH, Selb, Germany) was used for thermal analysis. The samples were accurately weighed and placed in an alumina crucible and heated from room temperature to 250 °C with a heating rate of 10 k/min.

### 2.11. In Vitro Aerosol Performance

The in vitro aerosol delivery performance of the formulations was evaluated using a next-generation impactor (NGI, Logan Instruments Corp., Somerset, NJ, USA), and the tests were performed in strict accordance with the requirements of General Rule 0951 Determination of Aerodynamic Particle Size of Inhalation Preparations in Volume IV of the Pharmacopeia of the People’s Republic of China. Prior to each NGI test, the complete NGI apparatus was placed in an environment at 25 °C and 40% relative humidity for equilibration for at least 30 min. The critical flow condition was verified: the pressure ratio across the control valve (P3/P2) was confirmed to be ≤0.5 to ensure the formation of critical flow inside the valve before experimental measurement. For flow calibration, a TSI 4000 series flow meter was connected to the NGI pipeline to measure the actual flow rate. The test was initiated only after confirming that the full flow path of the impactor could stably maintain a flow rate of 60 L/min under the set vacuum degree; the flow rate was rechecked before each batch of experiments to ensure detection accuracy. In this study, size 3 gelatin capsules were used for powder loading, with each capsule accurately filled with approximately 10 mg of test powder. A total of 10 capsules were used for a single NGI test to ensure that the drug mass deposited at each stage was sufficient for accurate HPLC quantification. All aerodynamic performance tests were performed in triplicate using independently prepared powder batches (*n* = 3), and the results are expressed as mean ± standard deviation (Mean ± SD). All collection cups were coated with a methanol solution containing 5% (*w*/*w*) glycerol, and the apparatus was assembled after the methanol was completely volatilized to avoid particle bounce and re-entrainment. The collection cups were placed in the tray, the lid was closed, and the handle was pressed down to seal the apparatus. A total of 15 mL of ultrapure water was added to the central collection cup of the pre-separator insert, and the pre-separator body was installed and fastened. An L-shaped connecting tube was inserted into the inlet end of the pre-separator, and a suitable mouthpiece adapter was installed at the other end of the L-shaped connecting tube. The filled capsules were loaded into the inhaler and pierced, then quickly connected to the NGI induction port. The vacuum pump was activated for 4 s to complete the dispersion and delivery of the powder. An interval of 30 s was set between two consecutive suctions to avoid interference with the results from residual powder in the device. After 10 suctions, the drug deposited in the drug delivery device, connecting tube, pre-separator and collection cups at each stage was rinsed with ultrapure water and brought to a constant volume. After quantification by HPLC, the mass of drug deposited at each site was calculated.

Fine particle fraction (FPF), mass median aerodynamic particle diameter (MMAD), geometric standard deviation (GSD), and emitted fraction (EF) can be obtained based on weight measurement and HPLC results.

The emitted fraction (EF) was calculated according to the formula:

EF (%) = [total loaded powder mass (gravimetric method)–retained dose in capsules (gravimetric method)]/total loaded powder mass (gravimetric method) × 100%.

The total loaded dose refers to the total mass of powder in 10 capsules before the test, accurately weighed by an analytical balance in milligrams (mg). After weighing, the powder was evenly divided into 10 capsules for the subsequent next-generation impactor (NGI) experiment. The retained dose is calculated by the differential weighing method for the residual powder mass in the capsules after inhalation at the end of the NGI test. Accordingly, both the numerator and the denominator in the EF calculation are actually based on the same powder mass basis.

The fine particle fraction (FPF) was calculated as follows:

FPF (%) = mass of drug with aerodynamic diameter ≤ 5 µm/total emitted drug mass × 100%

The mass of drug with aerodynamic diameter ≤ 5 µm was obtained by interpolation based on the nominal cut-off diameters of each NGI stage and the HPLC quantitative results of each collection cup. The total emitted drug mass was defined as the sum of drug deposition in the induction port, pre-separator and collection cups at all stages.

For the mass median aerodynamic diameter (MMAD) and geometric standard deviation (GSD), the cumulative mass-particle size distribution curve of the drug was plotted based on the drug deposition mass at each collection cup determined by HPLC, and the values were calculated by the log-probability regression method. MMAD corresponds to the aerodynamic diameter at which the cumulative drug mass reaches 50%, while GSD is the ratio of the particle diameter at 84.13% of the cumulative distribution to that at 50%, which reflects the width of the particle size distribution.

### 2.12. High-Performance Liquid Chromatography (HPLC)

The content of molnupiravir was measured by a high-performance liquid chromatography system with ultraviolet detection (HPLC-UV, 1260 Infinity, Agilent Technologies, Santa Clara, CA, USA) with a C18 column (250 × 4.6 mm, 5 μm). Isocratic elution was performed using acetonitrile and water in a ratio of 80 to 20. The column temperature was set to 30 °C, sample injection volume to 10 μL, flow rate to 0.8 mL/min, running time to 10 min and the UV detector at 240 nm. Samples containing molnupiravir were filtered through a 0.22 μm nylon membrane before analysis.

### 2.13. In Vitro Dissolution

The dissolution of samples was determined in artificial lysosomal fluid (ALF) and Gamble’s solution (GMB). GMB mimics deep interstitial fluid in the lung under normal health with a pH of 7.3 [32], while ALF mimics the intracellular state of lung cells after phagocytosis with a pH of 4.5 [33]. The sample (250 mg) was added to 10 mL GMB and ALF (*n* = 3) at a stirring speed of 50 rpm and a temperature of 37 °C. A total of 1 mL of solution was taken at 1, 2, 5, 10, 15, 20, 30, 45, and 60 min for subsequent analysis, and 1 mL of GMB or ALF prewarmed at 37 °C was added after sampling. Based on the experimental conditions presented above, the in vitro dissolution assay in this study did not maintain sink conditions. Accordingly, the obtained dissolution rates represent the apparent performance exhibited under the specific dose and specific medium volume, and the conclusions cannot be directly generalized to describe the intrinsic dissolution rate of the drug itself.

### 2.14. Process Evaluation via Machine Learning

To examine the impact of various factors such as concentration, feeding rate, and nebulization pressure on particle size and flowability, we employed different machine learning approaches, including explicit quadratic regression models (Poly2_Linear and Poly2_Ridge), neighbor-based and kernel-based models (k-nearest neighbors, support vector regression with a radial basis function kernel, Gaussian process regression), tree-based and ensemble learning models (decision tree, random Forest, gradient boosting, XGBoost 3.2.0 and HistGradientBoosting). The operational factors were treated as independent variables or features, while each *D*_V_, *D*_ae_, HR, and CI, served as the dependent variable or target in our analysis. These technical frameworks include explicit nonlinear regression, local similarity-based learning, kernel-based regression, and tree-based ensemble learning, which enable a comprehensive evaluation of performance discrepancies among various methods to predict the key quality attributes (KQAs) of inhaled formulations.

As shown in Table S3, 33 samples prepared by SFD methods were applied for ML analysis. Concentration (C), nebulization pressure (NP), and feeding rate (FR) were used as predictors. Meanwhile, D_v_, D_ae_, Carr index (CI) and Hausner ratio (HR) were the four regression targets. A single hold-out split would provide an unstable estimate for *n* = 33, so repeated nested cross-validation was used in this study. The outer loop was RepeatedKFold with five folds and ten repeats (random_state = 42), which yielded 50 outer test-fold evaluations for every model-target combination. Depending on the fold, 26 observations were used for training and seven for testing. Each observation therefore served as held-out test data in every repeat. StandardScaler was embedded within each scikit-learn 1.8.0 Pipeline, so the scaling parameters were estimated from the applicable training data only. Within every outer training fold, models with tunable hyperparameters were optimized using shuffled three-fold KFold GridSearchCV (random_state = 42), with R^2^ as the inner scoring criterion and refit = True. The selected configuration was refit on the complete outer training fold and evaluated on the untouched outer test fold. Poly2_Linear had no tuned hyperparameters and was fitted directly within each outer training fold. R^2^, RMSE, and MAE were calculated for each outer test fold and summarized as mean ± standard deviation across the 50 outer-fold scores. Mean outer-fold R^2^ was the primary ranking criterion, with RMSE and MAE used as secondary criteria. Although the nested design prevented preprocessing and hyperparameter selection from using outer-test information, the sample size remained small; each outer test fold contained only six or seven observations, and the large standard deviations indicated substantial split-to-split uncertainty. Therefore, the analysis was described as exploratory internal validation and made no claim of external generalizability.

## 3. Results and Discussion

### 3.1. Characterization of Molnupiravir Microparticles Prepared by Sfd

#### 3.1.1. Size Distribution and Fluidity of Molnupiravir Inhalable Powder

**Figure 1:**
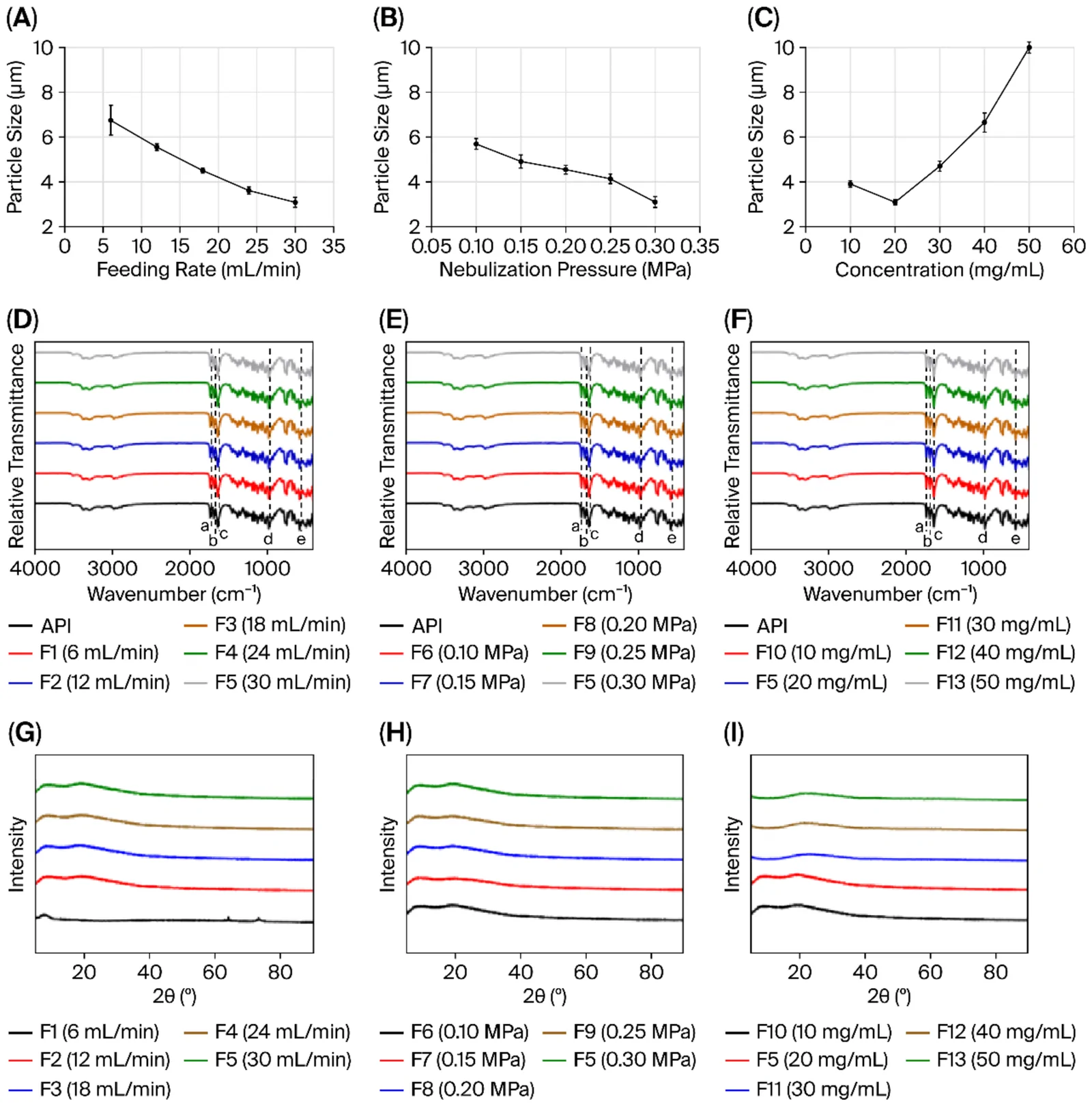
The relationship between particle size (*D*_v_) and (**A**) feeding rate (5–30 mL/min); (**B**) nebulization pressure (0.1–0.3 MPa); (**C**) concentration (10–50 mg/mL). (**D**–**F**) FTIR spectrum (characteristic absorption peaks: (a) 1730 cm^−1^, νC=O; (b) 1684 cm^−1^, νC=O; (c) 1632 cm^−1^, νC=C; (d) 979 cm^−1^, γC-H; (e) 583 cm^−1^, γO-H; (**G**–**I**) XRD results.

The particle size distribution of as-prepared samples is summarized in Table 1. The *D*_V_ value of the powders produced via SFD (except F13) ranged from 1 to 7 μm. Meanwhile, the approximate theoretical value of *D*_ae_ calculated by Equation (6) was less than 1 μm (except F13), which could be attributed to the low density of the powders (Table S2). Figure 1A–C illustrate the *D*_V_ value of the powders prepared under varying feeding rate, nebulization pressure, and API concentration. The value of *D*_V_ decreased from 6.75 ± 0.67 μm (F1) to 3.08 ± 0.24 μm (F5) when increasing the feeding rate from 6 to 30 mL/min. Similarly, elevating the nebulization pressure from 0.1 MPa to 0.3 MPa caused a moderate reduction in particle size (*D*_V_) from 5.68 ± 0.26 μm (F6) to 3.08 ± 0.24 μm (F5). Furthermore, the *D*_V_ value dramatically improved by 224% (from 3.08 ± 0.24 μm to 9.98 ± 0.24 μm) when the concentration changed from 20 mg/mL to 50 mg/mL. Increasing the feeding rate and nebulization pressure could facilitate the formation of initial nebulized droplets with smaller diameters during the spray-freezing process, ultimately reducing particle size [34]. In comparison, increasing the concentration led to higher solute content and lower water content in the initial aerosol droplets, resulting in an enlargement of the particle size after freeze-drying [35].

The flowability of inhalable powders significantly affects their dispersion and deposition behavior in the lung [36,37]. Carr index (CI) and Hausner ratio (HR), which are calculated based on the bulk and tapped density, were used to evaluate powder fluidity. Generally, the powder would exhibit better flowability when the CI was less than 20% [38]. In addition, according to Geldart fluidization classification, the powder can be considered as fluidizable when the HR was below 1.25 [39]. As depicted in Table S2, the bulk and tapped density of particles prepared via SFD were less than 13 mg/cm^3^ and 15 mg/cm^3^, respectively. Moreover, the CI and HR values of all particles were below 20% and 1.25, indicating excellent flowability and dispersibility of the SFD powders.

#### 3.1.2. Ftir and Xrd of Molnupiravir Inhalable Powder

The FTIR of molnupiravir API and SFD-prepared samples are shown in Figure 1D–F. The results show that the characteristic absorption peaks corresponding to the core functional groups of molnupiravir were well retained in all SFD samples, and no obvious generation of new peaks or disappearance of characteristic peaks was observed, suggesting that the SFD process did not disrupt the chemical skeleton of the drug. Specifically, the bands at 1730 cm^−1^ and 1684 cm^−1^ correspond to the stretching vibration of C=O bonds, whereas the band at 1632 cm^−1^ originates from the stretching vibration of the C=C double bond. In addition, the bands at 979 cm^−1^ and 583 cm^−1^ were related to the bending vibration of the C-H and O-H bonds, respectively. The XRD patterns of molnupiravir API and SFD powders are shown in Figure 1G–I and Figure S1, respectively. The absence of sharp diffraction peaks within the tested range demonstrates the amorphous state of all SFD samples. Previous studies have demonstrated that amorphous particles can improve the apparent solubility of drugs and increase the bioavailability, which is beneficial for pulmonary drug delivery [40,41].

#### 3.1.3. In Vitro Aerodynamics of Inhalable Powder

**Figure 2:**
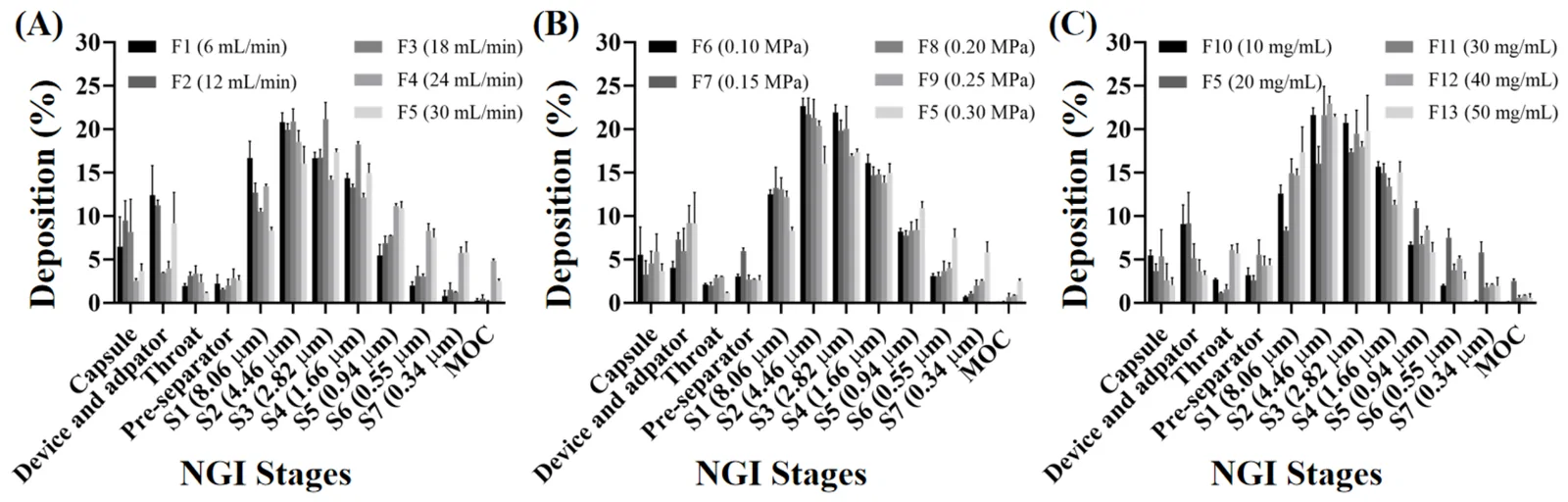
In vitro simulated lung deposition (**A**–**C**).

The in vitro aerodynamic performance of the inhalable powders was characterized by emitted fraction (EF), fine particle fraction (FPF), mass median aerodynamic diameter (MMAD) and geometric standard deviation (GSD). These four parameters are summarized in Table 1. The EF values of all samples exceeded 90%. Meanwhile, the FPF values were all higher than 50%, and MMAD values ranged from 3 to 5 μm, demonstrating that these samples were suitable for pulmonary drug delivery. In addition, the GSD values were below 3, except for F4 (3.58 ± 0.67), which indicates a relatively narrow particle size distribution. Figure 2 presents in vitro stimulated lung deposition results measured by NGI. Notably, the loss of dry powder particles across four stages (capsule, device and adapter, throat and pre-separator) was below 15% (Figure 2A–C), which reveals good deposition behavior in the deep lung regions.

Among all samples, F5 exhibited the highest FPF (70.19 ± 2.06%) and the smallest MMAD (2.80 ± 0.16 μm). FPF represents the proportion of fine particles (1–5 μm) within the overall particle size distribution. Notably, particles within this specific size range (1–5 μm) are regarded as optimal for inhalation. In addition, particles with lower MMAD tend to deposit deeply in the lung. As particles with low MMAD and high FPF could achieve better pulmonary delivery, which reflects good aerodynamic performance [42], F5 was selected for subsequent evaluation.

#### 3.1.4. Significance Evaluation of the Process Parameters via Machine Learning

Based on the data presented in Table S3, different machine learning approaches were employed to identify which process parameters influence the production process significantly. As shown in Table S4 and Figure S2, the predictive capability of different models exhibited marked differences across four critical quality attributes. Overall, all models, especially the explicit quadratic regression models (Poly2_Linear and Poly2_Ridge) and kernel-based models (Gaussian process regression, GPR), achieved a higher prediction performance on *D_V_* and *D_ae_* than on CI and HR. For the prediction of *D_V_*, GPR could attain a higher R^2^ value (0.782 ± 0.282), reflecting its optimal fitting capacity for the nonlinear correlation among *D_V_* and operation factors. For error metrics, Poly2_Linear had the minimum root mean square error (RMSE, 0.503 ± 0.309) and mean absolute error (MAE, 0.405 ± 0.190). In comparison, Poly2_Linear exhibited the optimal predictive capability on *D_ae_*. The values of R^2^, RMSE, and MAE were 0.744 ± 0.234, 0.057 ± 0.035 and 0.045 ± 0.021, respectively.

**Figure 3:**
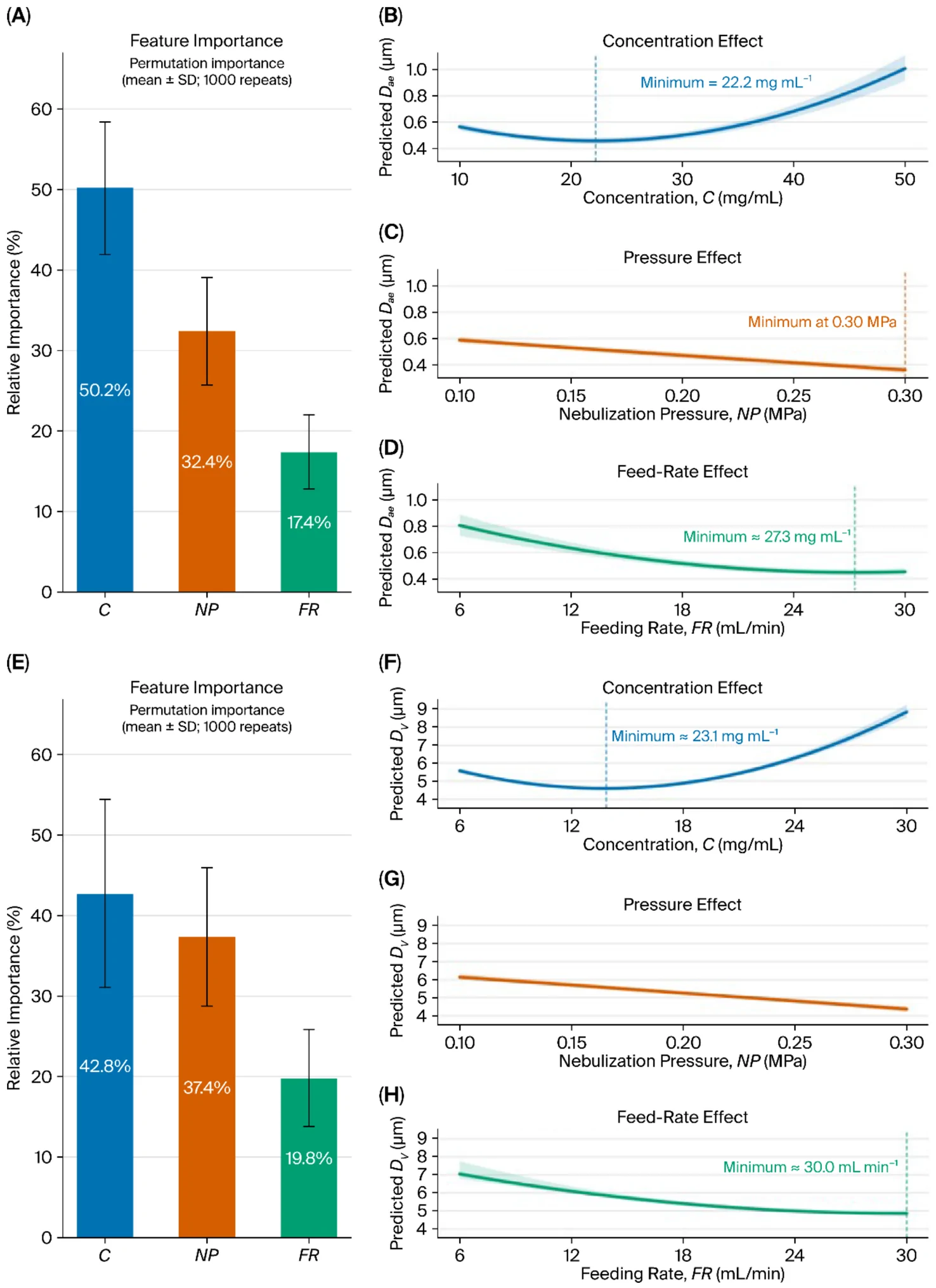
(**A**–**D**) Feature importance and effect trends of process parameters on D_ae_ based on the Poly2_Linear model. (**A**) Permutation importance of C, NP, and FR; (**B**–**D**) their corresponding univariate effects on D_ae_. (**E**–**H**) Feature importance and effect trends of process parameters on D_V_ based on the GPR model. (**E**) Permutation importance of C, NP, and FR; (**F**–**H**) their corresponding univariate effects on D_V_. Error bars represent standard deviations, shaded areas indicate 95% pointwise confidence intervals, and dashed lines mark the predicted minima. (FR = feeding rate; C = concentration; NP = nebulization pressure).

Figure 3 depicts the impact of process parameters on particle size using Poly2_Linear (Figure 3A–D) and GPR (Figure 3E–H) models. Permutation importance analysis showed that the contributions of concentration (*C*), nebulization pressure (*NP*), and feeding rate (*FR*) to *D_ae_* were 50.2%, 32.4%, and 17.4%, respectively. Meanwhile, their contributions to *D_V_* were 42.8%, 37.4%, and 19.8%. The importance ranking for both particle size metrics was *C* > *NP* > *FR*. *C* exhibited a U-shaped effect on both *D_ae_* and *D_V_*, with minimum predicted values occurring at approximately 22.2 and 23.1 mg/mL, respectively. Increasing the value of *NP* leads to a reduction in particle-size metrics. *FR* showed an initial decrease followed by a slight increase in *D_ae_*, with a minimum at approximately 27.3 mL/min, whereas its effect on *D_V_* gradually plateaued at higher feed rates. These results indicate that concentration was the dominant factor affecting particle size, followed by nebulization pressure and feeding rate.

### 3.2. Comparison of Molnupiravir Api, Sfd-F5 and Jm Samples

#### 3.2.1. Sem, Psd, Ftir and Xrd Properties

**Figure 4:**
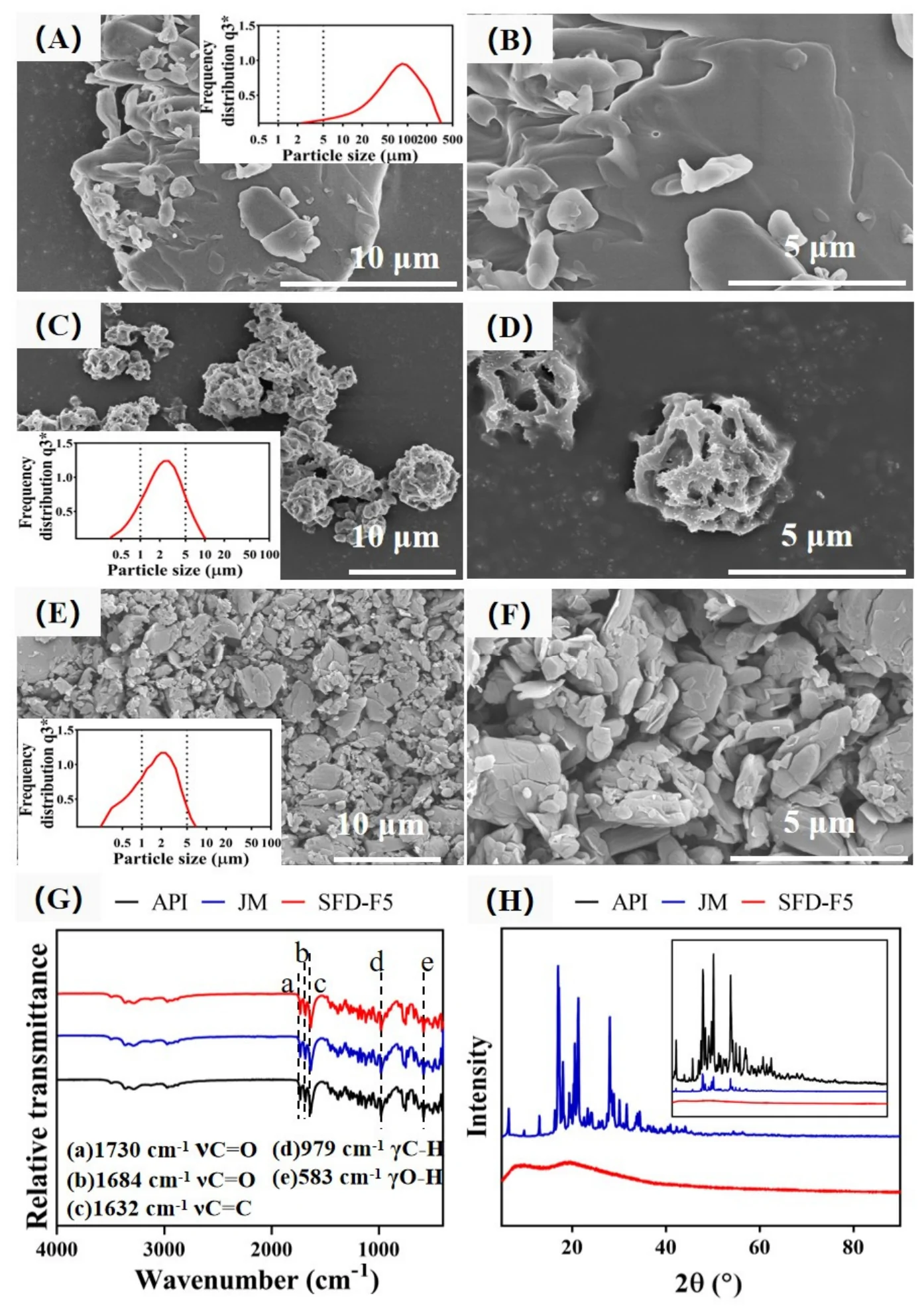
Morphology and particle size distribution of (**A**,**B**) API; (**C**,**D**) SFD-F5; (**E**,**F**) JM powders. (**G**) FTIR and (**H**) XRD of API, SFD-F5 and JM powders.

Figure 4 reveals the SEM, PSD, FTIR, and XRD results of molnupiravir API and the powders prepared by SFD (labeled as SFD-F5) and JM (labeled as JM) methods. As illustrated in Figure 4A,B, molnupiravir API displays a flaky morphology with a larger particle size (66.08 ± 2.48 μm), which is not suitable for pulmonary drug delivery. In contrast, Figure 4C,D reveal that the powders prepared by the SFD possess a near-spherical morphology, porous structure, smaller particle size, and narrower particle size distribution. The formation of these powders could be attributed to the droplets being frozen instantaneously through contact with liquid nitrogen to form spherical ice crystals during the SFD process. Subsequently, the moisture sublimated from the spherical ice crystals during the freeze-drying process, leading to the generation of a spherical and porous structure. The porous structure could increase the surface area of the particles and enhance solubility, which is beneficial for improving drug bioavailability. Furthermore, the spherical structure can be easily fluidized to generate stable aerosols in airflow [43], which is suitable for pulmonary drug delivery [44]. The powders directly jet-milled from the API consisted of irregular flakes. The particle size was dramatically reduced from 66.08 ± 2.48 μm (API) to 1.90 ± 0.04 μm (Figure 4E,F). In Figure 4G, all samples exhibited the same infrared spectrum signature absorption bands, indicating that the chemical stability was not changed during the JM and SFD processes. In Figure 4H, the JM sample reveals distinct diffraction peaks, proving its well-defined crystalline structure. Nevertheless, the diffraction peak intensities are markedly lower than those of API, which might be caused by the reduction in particle size during the JM process.

#### 3.2.2. Thermal Analysis and Stability

**Figure 5:**
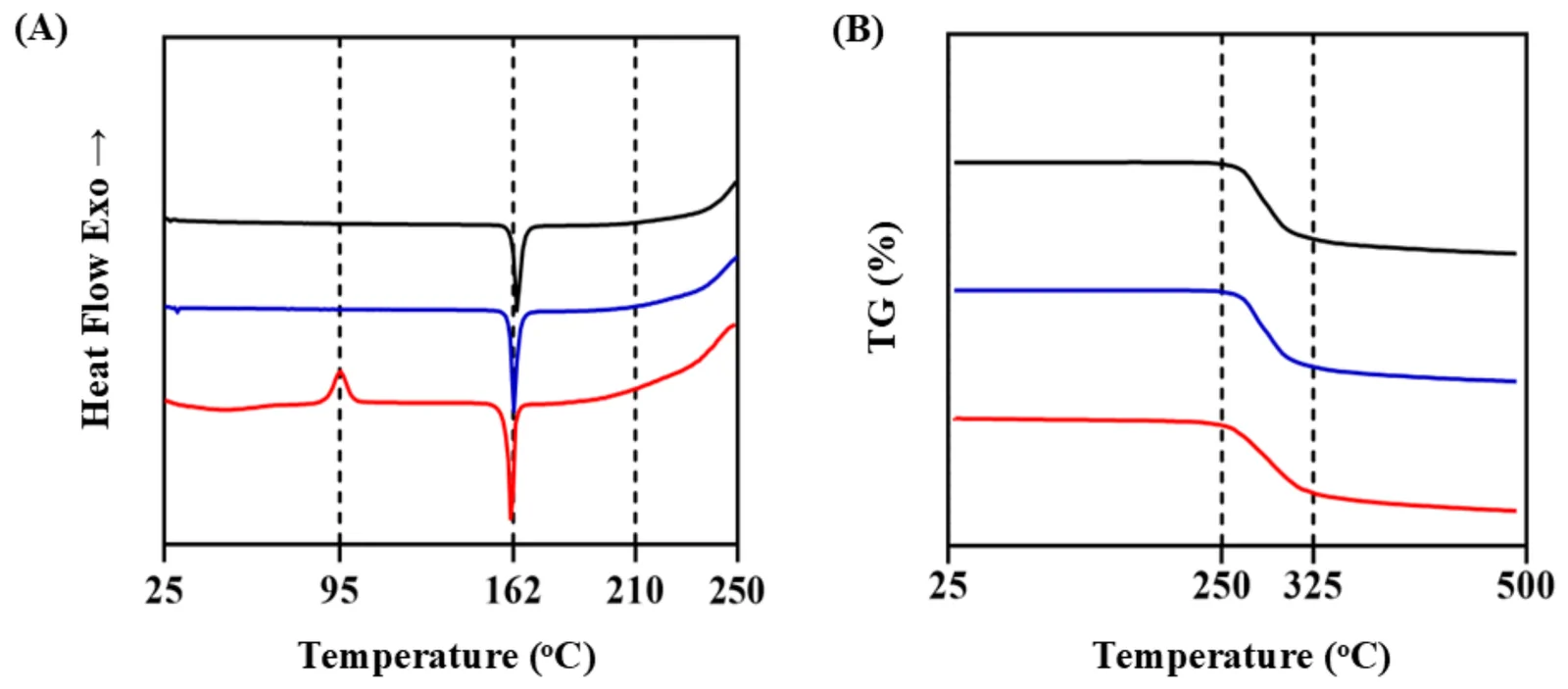
DSC (**A**) and TG (**B**) results of API, SFD-F5 and JM powders.

The thermal properties of molnupiravir API, JM and SFD-F5 powders were characterized via DSC (Figure 5A) and TG (Figure 5B). As depicted in Figure 5A, all samples exhibited a distinct endothermic peak corresponding to the melting behavior of crystalline molnupiravir at 162 °C. When the temperature exceeded 200 °C, the pronounced exothermic behavior corresponded to the degradation of molnupiravir under elevated temperature conditions. In contrast to JM and API, SFD-F5 showed a distinct exothermic peak at 95 °C, which is attributed to the temperature-induced crystallization behavior of amorphous molnupiravir [45]. In Figure 5B, the rapid mass loss occurring between 250 °C and 375 °C originates from the oxidative decomposition and thermal degradation of molnupiravir. The residual mass of all samples remained nearly constant as the temperature rose from 325 to 500 °C, corresponding to the completion of the oxidative decomposition.

**Figure 6:**
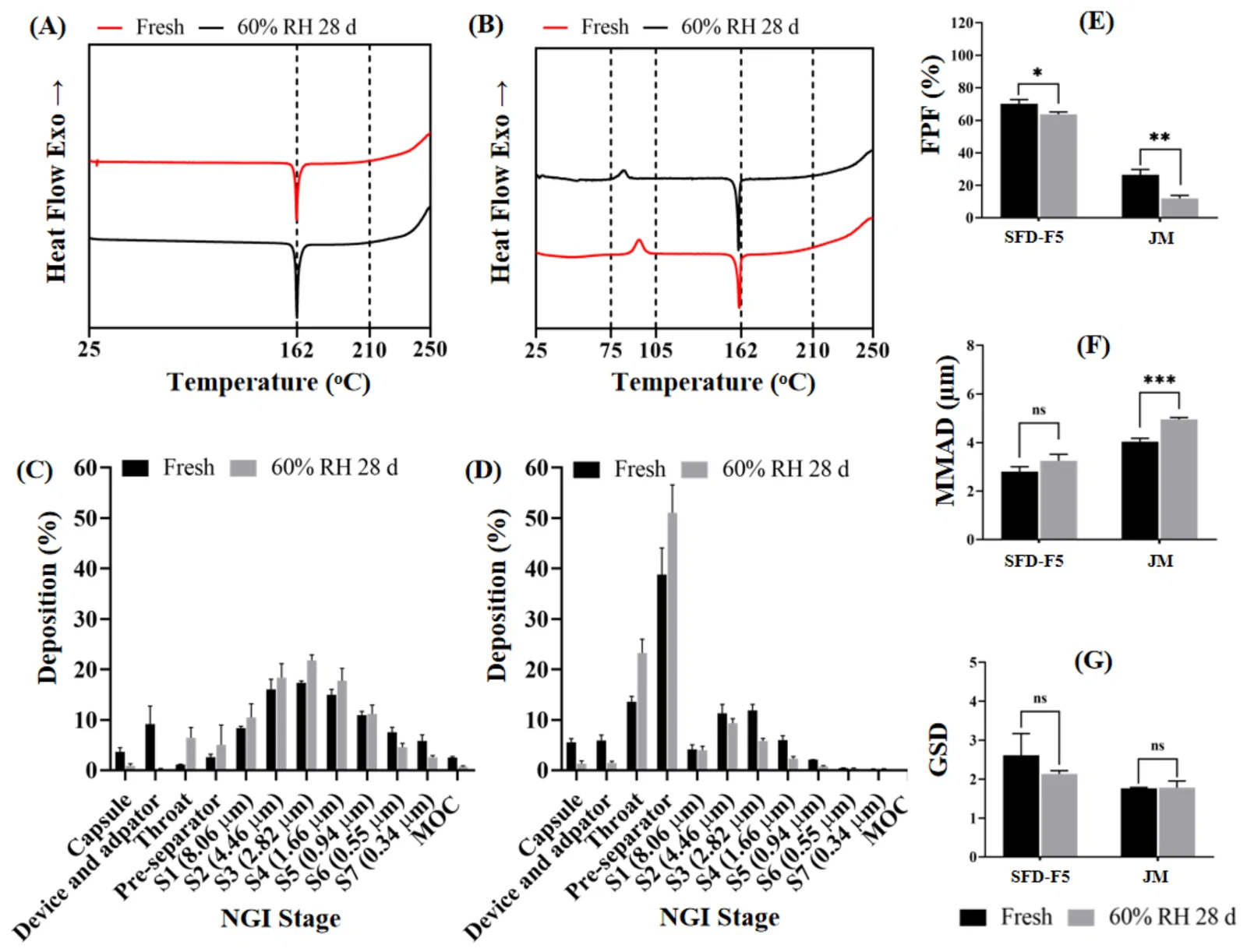
Stability evaluation of samples after 28 days of storage: DSC of (**A**) SFD-F5 and (**B**) JM; Simulated lung deposition of (**C**) SFD-F5 and (**D**) JM. Aerodynamics of SFD-F5 and JM: (**E**) FPF; (**F**) MMAD; (**G**) GSD [Significant difference: ns indicates no significant difference (*p* > 0.05); * indicates significant difference (*p* < 0.05); ** indicates very significant difference (*p* < 0.01); *** indicates extremely significant difference (*p* < 0.001)].

The SFD-F5 and JM samples were stored at 25 °C and 60% relative humidity for 28 days to preliminarily investigate the short-term storage behavior of the samples under this accelerated condition. As shown in Figure 6A, certain differences in crystallization characteristics were observed for SFD-F5 samples before and after storage, suggesting that the amorphous SFD-F5 powder may have undergone partial recrystallization with a decreased amorphous fraction under this humidity condition. This change is consistent with the inherent characteristics of the thermodynamically metastable state of amorphous drug systems. As shown in Figure 6B, no significant changes were observed in the endothermic and exothermic DSC curves of the JM sample after 28 days of storage, preliminarily indicating that its crystalline structure remained relatively stable under the experimental conditions of this study. It should be noted that the above results are only applicable to the 28-day storage period and the specific temperature and humidity conditions set in this study, and can only reflect the variation trend of the samples within this investigation period. The long-term physical stability of the powder, as well as its stability performance under a wider range of storage conditions, still needs to be further systematically investigated and verified. Regarding the in vitro aerodynamic performance after 28 days of storage, the deposition fractions of SFD-F5 at the throat and pre-separator stage changed slightly, rising from 1.22 ± 0.07% and 2.78 ± 0.52% to 6.42 ± 2.07% and 5.05 ± 3.92% (Figure 6C). In comparison, the deposition rates of JM powders deposited in the throat and pre-separator increased from 13.69 ± 1.05% and 39.00 ± 4.65% to 23.26 ± 2.75% and 51.07 ± 5.52%, respectively (Figure 6D). Furthermore, the aerodynamic performance was affected significantly by the humid environment (60% RH). After 28 days of storage, the FPF of SFD-F5 decreased slightly from 70.19 ± 2.06% to 63.36 ± 1.23%, whereas the FPF of JM declined drastically from 26.47 ± 2.68% to 11.95 ± 1.50% (Figure 6E). The MMAD value of SFD-F5 and JM powders (Figure 6F) increased slightly from 2.80 ± 0.16 μm and 4.04 ± 0.11 μm to 3.25 ± 0.22 μm and 11.95 ± 1.50 μm, respectively. The GSD of SFD-F5 decreased from 2.62 ± 0.45 to 2.14 ± 0.07, while the GSD of the JM sample increased from 1.77 ± 0.02 to 1.78 ± 0.14 (Figure 6G). The negative effect of humidity on the JM sample was more significant than that on SFD-F5, which can be ascribed to structural and morphological differences between the samples. The porous and near-spherical morphology of SFD-F5 could reduce the inter-particle contact area and cohesive interaction to a certain extent, which helped to alleviate the degradation of aerodynamic performance caused by particle agglomeration under humid conditions. In contrast, JM powders exhibited a flaky morphology with a larger inter-particle contact area, which made them more prone to particle agglomeration under moisture absorption conditions, thus leading to a more pronounced decline in aerosol performance [46,47].

#### 3.2.3. In Vitro Aerodynamic Performance

**Figure 7:**
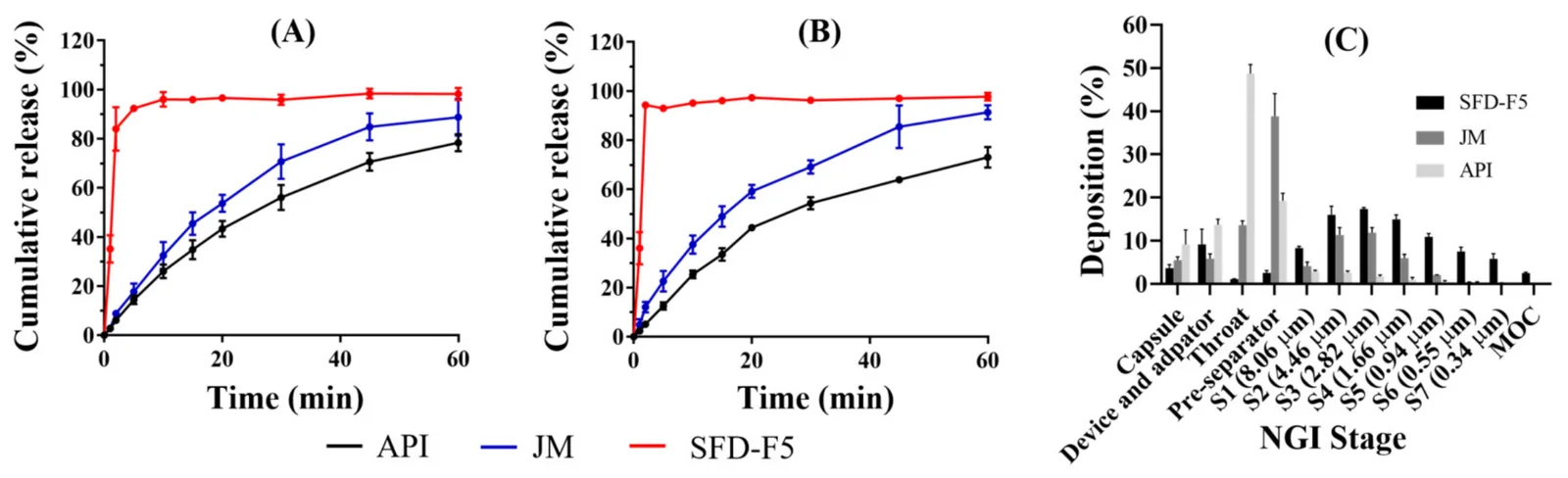
(**A**) Cumulative release curves in GMB; (**B**) Cumulative release curves in AlF; (**C**) Simulated lung deposition of SFD-F5, JM and API powders.

As summarized in Table 2, SFD-F5 revealed better in vitro aerodynamic performance due to the higher FPF (70.19 ± 2.06%) and lower MMAD (2.80 ± 0.16 μm). Figure 7A shows substantial deposition of JM powders at the throat (13.68 ± 1.05%) and pre-separator (39.00 ± 4.65%) stages, which were considerably higher than those of SFD-F5 (throat 1.13 ± 0.09%, pre-separator 2.59 ± 0.59%). The poor aerodynamic performance of the JM sample might be caused by the irregular flaky morphology, which hinders smooth fluidization under airflow. In contrast, the improved aerodynamic performance of SFD-F5 powders can be attributed to their lower density (Table S2), smaller *D*_ae_ (Table 1), as well as porous and near-spherical morphology (Figure 4C,D). Lower density and smaller *D*_ae_ contribute to the enhanced particle dispersion and fluidization in airflow [48]. Furthermore, the SFD-F5 particles could be transported faster and farther under the same conveying force due to the rough surface and the porous structure [49].

#### 3.2.4. Dissolution Rate Analysis

The dissolution behavior of molnupiravir powders was measured in GMB (Figure 7B) and ALF (Figure 7C) solutions. Generally, molnupiravir showed poor dissolution behavior in pure water (<30 mg/mL) at room temperature (25 °C). Notably, the cumulative dissolution rate of SFD-F5 powder reached 92.38 ± 0.54% within 5 min in GMB solution, which was substantially higher than those of JM (17.66 ± 3.38%) and API (14.43 ± 1.74%) samples. After 60 min, the cumulative dissolution rate of API and JM samples increased dramatically to 78.42 ± 3.44% and 88.72 ± 7.46%, respectively, which were still lower than that of SFD-F5 (97.89 ± 2.35%). In the ALF solution, the dissolution profiles of JM, SFD-F5, and API powders exhibited a similar tendency in GMB (Figure 6C). After 5 min in ALF solution, the cumulative dissolution rate of JM (22.58 ± 4.16%) and API (12.52 ± 1.49%) samples was extremely lower than that of SFD-F5 (94.81 ± 5.19%). After 60 min, around 97.71 ± 1.38% of SFD-F5 powders dissolved in ALF, which was still higher than that of JM (91.36 ± 2.84%) and API (73.04 ± 4.22%) powders.

The differences in dissolution rate can be attributed to the different physicochemical properties of the powders. The initial dissolution disparity relied on the particle morphology. According to the Noyes–Whitney equation, surface area crucially influences dissolution rate [50]. The porous structure of SFD-F5 particles significantly strengthens their interaction with the solvent, thereby greatly enhancing the dissolution rate. Meanwhile, after micronization by airflow, JM powders still maintained irregular plate-like morphology. Although particle size was reduced and surface area increased, these features contributed partially to solubility enhancement. Furthermore, amorphous particles are not constrained by lattice energy during dissolution, enabling faster dissolution in comparison with crystalline counterparts. Therefore, SFD-F5 achieved the highest dissolution rate owing to its smaller particle size, porous structure, larger surface area and amorphous state. It should be noted that the above inferences regarding contact area and specific surface area are based on the observation of particle morphology, and the actual specific surface area of the particles still needs to be further determined and verified by experiments such as the nitrogen adsorption method (BET).

## 4. Conclusions

In this study, the molnupiravir inhalable powders with near-spherical morphology and porous structure were controllably prepared via the spray freeze-drying (SFD) method. Compared with the jet-milled (JM) method, the SFD powders exhibited superior aerodynamic properties characterized by higher FPF and lower MMAD, making them ideal candidates for pulmonary delivery. After storage for 28 days at 25 °C and 60% RH, molnupiravir micropowder still showed good preliminary short-term storage behavior. In addition, ten regression models using repeated nested cross-validation were applied to evaluate the significance of process parameters. GPR achieved the highest mean outer R^2^ for D_V_ prediction (0.782 ± 0.282), whereas Poly2_Linear performed well for D_ae_ prediction (R^2^ = 0.744 ± 0.234). Permutation importance and univariate effect analyses showed that concentration was the dominant process factor affecting both particle size metrics, followed by nebulization pressure and feeding rate. Therefore, this study offers innovative ideas for the advancement of inhalable powders and shows the potential for lung infection treatment through pulmonary drug delivery.

## Figures and Tables

**Table 1:** Particle size distribution and aerodynamic parameters of SFD powders (Mean ± SD, *n* = 3).

No.	*D*_V_ (μm)	*D*_ae_ (μm)	EF (%)	FPF (%)	MMAD (μm)	GSD
F1	6.75 ± 0.67	0.70 ± 0.09	95.71 ± 2.62	54.10 ± 1.83	4.34 ± 0.09	2.24 ± 0.05
F2	5.56 ± 0.14	0.59 ± 0.02	93.10 ± 1.65	58.18 ± 0.82	3.93 ± 0.17	2.23 ± 0.17
F3	4.49 ± 0.07	0.42 ± 0.02	93.59 ± 2.99	64.04 ± 0.15	3.58 ± 0.07	2.03 ± 0.07
F4	3.61 ± 0.13	0.36 ± 0.02	97.66 ± 0.26	62.14 ± 0.37	3.01 ± 0.15	3.58 ± 0.67
F5	3.08 ± 0.24	0.34 ± 0.03	97.25 ± 0.74	70.19 ± 2.06	2.80 ± 0.16	2.62 ± 0.45
F6	5.68 ± 0.26	0.58 ± 0.01	95.28 ± 2.87	61.27 ± 1.27	3.83 ± 0.09	2.00 ± 0.03
F7	4.89 ± 0.03	0.58 ± 0.00	97.13 ± 1.74	57.53 ± 4.13	3.94 ± 0.31	2.08 ± 0.10
F8	4.54 ± 0.17	0.46 ± 0.02	96.34 ± 1.84	60.74 ± 1.12	3.75 ± 0.13	2.15 ± 0.19
F9	4.11 ± 0.21	0.49 ± 0.03	95.71 ± 1.71	59.73 ± 0.51	3.69 ± 0.01	2.32 ± 0.08
F10	3.89 ± 0.15	0.38 ± 0.00	96.18 ± 0.28	59.11 ± 1.09	3.96 ± 0.09	1.97 ± 0.02
F11	4.67 ± 0.09	0.41 ± 0.01	95.55 ± 2.94	57.23 ± 4.56	4.06 ± 0.29	1.98 ± 0.01
F12	6.64 ± 0.43	0.73 ± 0.06	97.67 ± 2.13	54.60 ± 1.05	4.04 ± 0.10	2.29 ± 0.06
F13	9.98 ± 0.24	1.11 ± 0.03	98.26 ± 0.84	53.64 ± 4.52	4.14 ± 0.29	2.24 ± 0.30

**Table 2:** Particle size distribution and in vitro aerodynamic parameters of API, JM, and SFD-F5 powders (Mean ± SD, *n* = 3).

Sample	D_50_ (μm)	D_V_ (μm)	EF (%)	MMAD (μm)	FPF (%)	GSD	Span
API	41.35 ± 2.70	66.08 ± 2.48	94.95 ± 2.21	5.71 ± 0.84	5.17 ± 1.80	2.20 ± 0.28	4.03 ± 0.24
JM	1.60 ± 0.02	1.90 ± 0.04	95.23 ± 0.42	4.04 ± 0.11	26.47 ± 2.68	1.77 ± 0.02	2.04 ± 0.07
SFD-F5	2.64 ± 0.02	3.08 ± 0.24	97.25 ± 0.74	2.80 ± 0.16	70.19 ± 2.06	2.62 ± 0.45	2.02 ± 0.19

## Data Availability

The data can be obtained from the corresponding authors based on reasonable request.

## Supplementary Materials

The following supporting information can be downloaded at: https://www.mdpi.com/article/10.3390/pharmaceutics18101290/s1, Figure S1: The XRD pattern of EIDD-2801 API; Figure S2: The R^2^ value and heatmap of machine models on *D_V_*, *D_ae_*, Carr index and Hausner ratio. Table S1: Preparation parameters of SFD particles; Table S2: The density and flowability of SFD particles; Table S3: The particle size and flowability data for machine learning; Table S4: The predictive capability of ten machine learning models on *D_V_*, *D_ae_*, Carr index and Hausner ratio; Table S5: Complete dose mass balance analysis for SFD-F5.

## Abbreviations

The following abbreviations are used in this manuscript:

SFD	Spray Freeze Drying
JM	Jet Milling
PDD	Pulmonary Drug Delivery
FPF	Fine Particle Fraction
MMAD	Mass Median Aerodynamic Diameter
AI	Artificial Intelligence
ML	Machine Learning
PSD	Particle Size Distribution
CI	Carr Index
HR	Hausner Ratio
SEM	Scanning Electron Microscope
FTIR	Fourier Translation Infrared Spectroscopy
TG	Thermogravimetric Analysis
DSC	Differential Scanning Calorimetry
NGI	Next Generation Impactor
GSD	Geometric Standard Deviation
EF	Emitted Fraction
HPLC	High-Performance Liquid Chromatography
ALF	Artificial Lysosomal Fluid
GMB	Gamble’s Solution
KNN	k-Nearest Neighbors
SVR_RBF	Support Vector Regression with a Radial Basis Function Kernel
GPR	Gaussian Process Regression
KQA	Key Quality Attribute(s)
CQA	Critical Quality Attribute(s)
C	Concentration
NP	Nebulization Pressure
FR	Feeding Rate
