# Supplementary material for: Preparation of Excipient-Free Molnupiravir Microparticles for Pulmonary Drug Delivery and Process Evaluation via Machine Learning
Source: Pharmaceutics. 2026 Oct 10;18(10):1290. doi: 10.3390/pharmaceutics18101290 (PMC13708680; doi:10.3390/pharmaceutics18101290)
Supplement: Supplementary file 1 [file pharmaceutics-18-01290-s001.zip › pharmaceutics-4502603-supplementary.pdf]

Preparation of excipient-free Molnupiravir microparticles for pulmonary drug delivery, and process evaluation via machine learning

Juncheng Hu[1,#], Yucheng Huang[2,#], Jiahao Luo[2], Jiayi Wang[2], Haoyang Chen[2], Minghao Lian[2],

Ning Xue[3], Xiang Luo[4] , Kaiqi Shi[5], Bin Dong[2,6*], Hanhan Li[7*]

[1] School of Artificial Intelligent Medicine, Guilin Medical University, Guilin 541199, PR China

[2] Department of Pharmaceutical and Environmental Engineering, China Pharmaceutical University, Nanjing 211198, PR China

[3] Department of Computer Science, University of Nottingham Ningbo China, Ningbo, Zhejiang, 315100, China

[4] Department of Chemical and Environmental Engineering, The University of Nottingham Ningbo China, Ningbo 315100, PR China

[5] Suzhou Inhal Pharma Co., Ltd, Suzhou, Jiangsu, 215000, PR China

[6] Engineering Research Center for Smart Pharmaceutical Manufacturing Technologies, Ministry of Education, China Pharmaceutical University, Nanjing, 210009, PR China

[7] School of Basic Medicine and Clinical Pharmacy, China Pharmaceutical University, Nanjing 21198, PR China

* Corresponding author: bin.dong@cpu.edu.cn (Bin Dong); 1020192600@cpu.edu.cn (Hanhan Li)

[#] Juncheng Hu and Yucheng Huang have contributed equally.

Table S1. Preparation parameters of SFD particles.

No.	Material concentration (mg/mL)	Nebulization pressure (MPa)	Feeding rate (mL/min)
F1	20	0.30	6
F2	20	0.30	12
F3	20	0.30	18
F4	20	0.30	24
F5	20	0.30	30
F6	20	0.10	30
F7	20	0.15	30
F8	20	0.20	30
F9	20	0.25	30
F10	10	0.30	30
F11	30	0.30	30
F12	40	0.30	30
F13	50	0.30	30

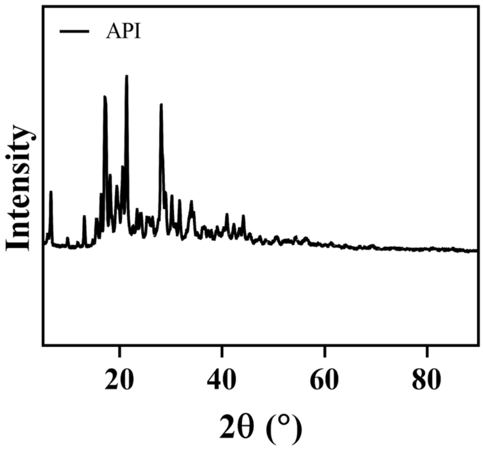

Figure S1. The XRD pattern of EIDD-2801 API.

Table S2. The density and flowability of SFD particles (Mean±SD, *n*=3).

No.	Bulk density (mg/cm^3)	Tap density (mg/cm^3)	Carr index (%)	Hausner ratio (/)
F1	9.38±0.45	10.68±0.85	12.05±2.82	1.14±0.04
F2	9.16±0.53	11.21±0.58	18.30±2.04	1.22±0.03
F3	10.35±0.31	12.85±1.10	19.13±5.83	1.24±0.09
F4	9.21±0.61	10.63±1.18	13.11±4.17	1.15±0.05
F5	10.08±0.98	11.91±1.20	15.32±1.22	1.18±0.02
F6	11.64±0.47	13.39±0.46	13.06±0.57	1.15±0.01
F7	11.42±0.77	14.17±0.95	19.38±3.08	1.24±0.05
F8	8.88±0.48	10.47±0.65	15.13±4.50	1.18±0.06
F9	12.11±0.64	14.04±0.58	13.75±2.64	1.16±0.04
F10	8.41±0.26	9.85±0.63	14.49±2.76	1.17±0.04
F11	10.91±0.19	13.40±0.84	18.46±3.71	1.23±0.06
F12	10.71±1.21	12.05±1.87	10.72±3.72	1.12±0.05
F13	10.06±0.05	12.31±0.20	18.28±1.67	1.22±0.03

Table S3. The particle size and flowability data for machine learning (Mean±SD, *n*=3).

No.	Concentration (*C*, mg/mL)	Nebulization Pressure (*NP*, MPa)	Feeding Rate (*FR*, mL/min)	D_V （μm）	D_{ae} （μm）	Carr index (%)	Hausner ratio
1	10	0.1	18	7.64±0.76	0.83±0.10	17.46±3.64	1.21±0.05
2	10	0.1	24	6.72±0.12	0.73±0.02	21.71±5.76	1.28±0.09
3	10	0.1	30	6.41±0.19	0.71±0.03	20.90±2.49	1.26±0.04
4	10	0.2	18	5.84±0.11	0.63±0.02	14.36±7.55	1.17±0.11
5	10	0.2	24	5.26±0.02	0.59±0.00	23.81±2.06	1.31±0.03
6	10	0.2	30	4.84±0.02	0.53±0.00	13.77±7.33	1.17±0.10
7	10	0.3	18	4.43±0.09	0.49±0.01	18.28±8.33	1.23±0.13
8	10	0.3	24	4.01±0.09	0.44±0.01	22.14±2.58	1.29±0.04
9	10	0.3	30	3.85±0.04	0.38±0.00	14.49±2.76	1.17±0.04
10	20	0.1	18	6.02±0.16	0.66±0.02	17.14±7.78	1.21±0.11
11	20	0.1	24	5.69±0.07	0.61±0.01	17.14±4.07	1.21±0.06
12	20	0.1	30	5.03±0.07	0.58±0.01	13.06±0.57	1.15±0.01
13	20	0.15	30	4.89±0.03	0.58±0.00	19.38±3.08	1.24±0.05
14	20	0.2	18	5.01±0.26	0.56±0.03	22.06±6.59	1.29±0.11
15	20	0.2	24	4.88±0.12	0.54±0.02	19.39±1.05	1.24±0.02
16	20	0.2	30	4.54±0.17	0.46±0.02	15.13±4.50	1.18±0.06
17	20	0.25	30	4.11±0.21	0.49±0.03	13.75±2.64	1.16±0.04
18	20	0.3	6	6.75±0.67	0.70±0.09	12.05±2.82	1.14±0.04
19	20	0.3	12	5.56±0.14	0.59±0.02	18.30±2.04	1.22±0.03
20	20	0.3	18	3.66±0.15	0.42±0.02	19.13±5.83	1.24±0.09
21	20	0.3	24	3.53±0.15	0.36±0.02	13.11±4.17	1.15±0.05
22	20	0.3	30	3.08±0.24	0.34±0.03	15.32±1.22	1.18±0.02
23	30	0.1	18	5.88±0.25	0.64±0.03	17.25±1.61	1.21±0.02
24	30	0.1	24	5.89±0.31	0.64±0.04	17.91±6.23	1.22±0.10
25	30	0.1	30	5.12±0.08	0.56±0.01	15.76±5.84	1.19±0.08
26	30	0.2	18	4.72±0.11	0.50±0.01	10.07±1.01	1.11±0.01

27	30	0.2	24	4.32±0.10	0.49±0.01	22.73±3.94	1.30±0.06
28	30	0.2	30	4.14±0.02	0.46±0.00	21.81±4.26	1.28±0.07
29	30	0.3	18	3.93±0.05	0.44±0.01	23.81±5.41	1.32±0.10
30	30	0.3	24	3.80±0.05	0.41±0.01	21.79±6.60	1.28±0.11
31	30	0.3	30	3.52±0.07	0.41±0.01	18.46±3.71	1.23±0.06
32	40	0.3	30	6.64±0.43	0.73±0.06	10.72±3.72	1.12±0.05
33	50	0.3	30	9.98±0.24	1.11±0.03	18.28±1.67	1.22±0.03

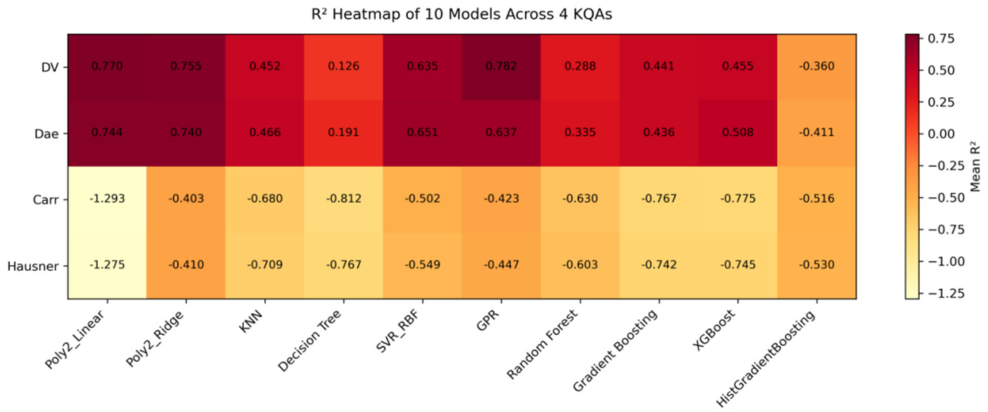

Figure S2. The R^2 value and heatmap of machine models on D_V, D_{ae}, Carr index and Hausner ratio

T

Table S4. The predictive capability of ten machine learning models on D_V, D_{ae}, Carr index and Hausner ratio

Target	Model	R^2	RMSE	MAE
D_V	GPR	0.782 ± 0.282	0.534 ± 0.444	0.403 ± 0.260
	Poly2_Linear	0.770 ± 0.204	0.503 ± 0.309	0.405 ± 0.190
	Poly2_Ridge	0.755 ± 0.279	0.522 ± 0.386	0.419 ± 0.265
	SVR_RBF	0.635 ± 0.347	0.695 ± 0.513	0.524 ± 0.321
	XGBoost	0.455 ± 0.603	0.824 ± 0.647	0.541 ± 0.375
	KNN	0.452 ± 0.470	0.891 ± 0.692	0.596 ± 0.406
	Gradient Boosting	0.441 ± 0.579	0.845 ± 0.639	0.556 ± 0.363
	Random Forest	0.288 ± 0.563	0.982 ± 0.666	0.650 ± 0.376
	Decision Tree	0.126 ± 0.731	1.037 ± 0.640	0.729 ± 0.376
	HistGradientBoosting	-0.360 ± 0.641	1.379 ± 0.583	1.083 ± 0.341
D_{ae}	Poly2_Linear	0.744 ± 0.234	0.057 ± 0.035	0.045 ± 0.021
	Poly2_Ridge	0.740 ± 0.274	0.058 ± 0.042	0.047 ± 0.029
	SVR_RBF	0.651 ± 0.327	0.074 ± 0.056	0.056 ± 0.035
	GPR	0.637 ± 0.295	0.076 ± 0.054	0.055 ± 0.032
	XGBoost	0.508 ± 0.550	0.086 ± 0.070	0.056 ± 0.040
	KNN	0.466 ± 0.431	0.096 ± 0.075	0.066 ± 0.044
	Gradient Boosting	0.436 ± 0.540	0.093 ± 0.068	0.063 ± 0.040
	Random Forest	0.335 ± 0.490	0.105 ± 0.071	0.071 ± 0.040
	Decision Tree	0.191 ± 0.627	0.108 ± 0.058	0.078 ± 0.035
	HistGradientBoosting	-0.411 ± 0.641	0.151 ± 0.065	0.117 ± 0.039
Carr index	Poly2_Ridge	-0.403 ± 0.930	3.830 ± 0.736	3.237 ± 0.768
	GPR	-0.423 ± 1.059	3.841 ± 0.807	3.227 ± 0.816
	SVR_RBF	-0.502 ± 1.143	3.941 ± 0.868	3.308 ± 0.821
	HistGradientBoosting	-0.516 ± 1.107	3.923 ± 0.886	3.327 ± 0.822
	Random Forest	-0.630 ± 1.389	4.044 ± 0.986	3.399 ± 0.918
	KNN	-0.680 ± 1.386	4.101 ± 0.993	3.388 ± 0.847
	Gradient Boosting	-0.767 ± 1.246	4.250 ± 1.025	3.537 ± 0.918
	XGBoost	-0.775 ± 1.242	4.248 ± 1.009	3.574 ± 0.888
	Decision Tree	-0.812 ± 1.319	4.233 ± 1.158	3.550 ± 1.108
	Poly2_Linear	-1.293 ± 1.993	4.653 ± 1.713	3.755 ± 1.377
Hausner ratio	Poly2_Ridge	-0.410 ± 0.980	0.057 ± 0.011	0.048 ± 0.012
	GPR	-0.447 ± 1.150	0.057 ± 0.012	0.048 ± 0.012
	HistGradientBoosting	-0.530 ± 1.152	0.059 ± 0.013	0.050 ± 0.012
	SVR_RBF	-0.549 ± 1.331	0.059 ± 0.014	0.049 ± 0.013
	Random Forest	-0.603 ± 1.415	0.060 ± 0.014	0.050 ± 0.013
	KNN	-0.709 ± 1.500	0.061 ± 0.014	0.050 ± 0.013
	Gradient Boosting	-0.742 ± 1.385	0.062 ± 0.016	0.052 ± 0.014
	XGBoost	-0.745 ± 1.379	0.062 ± 0.015	0.052 ± 0.013
	Decision Tree	-0.767 ± 1.432	0.062 ± 0.017	0.051 ± 0.016
	Poly2_Linear	-1.275 ± 2.130	0.069 ± 0.026	0.055 ± 0.021

Table S5. Complete dose mass balance analysis for SFD-F5

**Formulation**	**SFD-F5**			**Deposition %**				
**Test**	**1**	**2**	**3**	**1**	**2**	**3**	**Mean**	**std**
Capsule/mg	2.3	3.7	2.4	2.34	3.80	2.52	2.89	0.78
Device and adapter/mg	8.1	11.8	9.0	8.26	12.11	9.46	9.94	1.95
Throat/mg	1.2	1.0	1.1	1.22	1.03	1.16	1.14	0.09
Pre-separator/mg	3.0	2.6	1.9	3.06	2.67	2.00	2.58	0.55
Stage 1/mg	7.9	8.0	8.3	8.06	8.21	8.73	8.33	0.35
Stage 2/mg	17.6	13.7	15.2	17.96	14.06	16.00	16.01	1.97
Stage 3/mg	17.3	17.0	16.2	17.65	17.45	17.03	17.38	0.34
Stage 4/mg	13.7	15.7	14.1	13.98	16.12	14.83	14.98	1.07
Stage 5/mg	11.4	9.9	10.4	11.63	10.16	10.94	10.91	0.73
Stage 6/mg	6.5	7.2	8.1	6.63	7.39	8.52	7.51	0.97
Stage 7/mg	6.7	4.4	5.8	6.84	4.52	6.10	5.82	1.17
MOC/mg	2.3	2.4	2.6	2.35	2.46	2.73	2.51	0.16
Recovered dose/mg	98.0	97.4	95.1					
Emitted dose/mg	99.3	98.4	97.3					
Loading dose/mg	101.6	102.1	99.7					
